# Unhealthful Habitations

**Published:** 1859-08

**Authors:** 


### HALL'S JOURNAL OF HEALTH.

OUR LEGITIMATE SCOPE IS ALMOST BOUNDLESS: FOR WHATEVER BEGETS PLEASURABLE AND HARMLESS FEELINGS, PROMOTES HEALTH; AND WHATEVER INDUCES DISAGREEABLE SENSATIONS, ENGENDERS DISEASE.

*We aim to show how Disease may be avoided, and that it is best, when sickness comes, to take no Medicine without consulting an educated Physician.*

VOL. VI.] AUGUST, 1859. [No. 8.

### UNHEALTHFUL HABITATIONS.

We have occasionally noticed some sharp contradictions of our views in some points, in papers which are in the main conducted with ability. Our readers are advised in all such cases to think for themselves, and to inquire if the person who calls a statement in question, is likely to have special means of information in regard to it. Persons sometimes think they know a thing is not so, from their not knowing that the opposite of it is true. A man who has lived from infancy on a small island, and has never seen any other land, may feel quite sure in his own mind, that it is the only land in the world, simply because he has never seen any other land. It is unwise to assert any thing to be true, until we know that its opposite is not true.

### BEST TOOTH WASH.

On one occasion, a correspondent of a water-cure journal inquired if a statement of ours was true, that washing the teeth with pure white soap had a tendency to prevent the collection of tartar on the teeth. The editor replied simply, "It is all fudge." He, perhaps, could not conceive how such a thing as common soft soap could keep the teeth clear of tartar accretions, which were so hard that a steel instrument is employed by dentists to remove them. He evidently did not know that recent chemical and microscopical investigations, carefully conducted with all the aids of dental science, had demonstrated that this tartar was the product of a living insect, upon which neither vinegar nor tobacco juice had any effect whatever, but which was instantly destroyed by soapsuds; and following up this fact, persons have kept their teeth perfectly clear of re-accumulations of tartar, by simply washing them with white soap and brush, night and morning. Now and then it will fail, because some tartar is made by an insect which is but little affected by soapsuds.

### PAPERED ROOMS.

On another occasion we stated that persons had been poisoned by occupying rooms covered with green paper. Shortly after a City paper contained a column or two attempting to throw ridicule on the statement, giving facts, as so stated, where persons had lived and slept in green-papered rooms for years in good health. Now we will give a fact which is indisputable:

In the Fall of 1858, a youth was laboring under symptoms of poisoning by arsenic. In spite of all treatment, the symptoms increased in severity for two months, when the patient was sent to the country, where he was speedily restored to health.

On returning home, he occupied the same apartment; and in a month was worse than before. Thinking that a cistern near a wall of the room might occasion the ailment, he was removed to another room for two weeks to afford an opportunity for making the necessary alterations, when he was returned to his old room, in apparent health. In three or four weeks the same symptoms returned, but with an aggravated degree of severity. It was then suggested that it might be the green paper on the wall which caused the illness. It was removed; paper of a different color was put on; and still occupying the same room, the patient recovered his health, and remained well.

It was from facts like these, reported in standard medical publications, we founded our article. It will readily occur to the reader, that paper may have so little green in it, that any ill effect on the health may not be appreciable for weeks, or months, or years; and then again, some constitutions are less amenable to the influences of green paper than others. We cannot undertake to hedge our Journal with provisos, and authorities and nice distinctions, else we should make it as dry as a bone and heavy as lead, and it would lose largely of its practicality. We prefer to present broad facts, with their general inferences. Those who are hypercritical and are fond of nice distinctions, had better procure a different kind of reading.

If green paper, under any circumstances, poisons the human system, it is better to lay it down as a broad fact for practical purposes, that green paper ought not to be put on the walls of rooms. If any one is disposed to experiment as to how much green in any given pattern can be used with impunity, we certainly have no objection; but for the general good, it is better to lay down the clear statement, "rooms ought not to be covered with green paper."

If the paper is well glazed, comparatively little injury may result, for then there is less fuz to fly about the room; but where the pattern is not glazed but is velvetty, and the figure standing out from the paper, it is impossible to escape the poisonous effects. A single hour's sitting in such a room has been known to nauseate a whole company. From one foot square of one of those tufted or flock green papers, thirty grains of the powder was scraped off and sent to a chemist; and the amount of solid arsenic in it was eleven grains—over one-third.

### USE THE SUNSHINE.

A New York merchant noticed in the course of years that every book-keeper that came to him got sick, however healthy he appeared on his arrival. One day it occurred to him all at once, that the room occupied was on the first floor, and was so situated that the sun never shone in it. He at once changed it for an upper story apartment, which freely admitted the sun light, with the result of healthy book-keepers ever after.

### SEE WHERE YOU BUILD.

A New Yorker built for himself a few years ago a splendid mansion. Not long after he moved into it several members of the family became sick; this continuing for months, it was remembered that the house had been built over an old drain, on a damp marshy spot, the emanations from which constantly rose through the cellar and passed up into every room of the building. He changed his residence, and his family regained their usual health.

The practical inference to be derived from these statements is, that considering it is impossible to cure any disease as long as the causes of that disease are in operation, if on moving into a room or house, or neighborhood, a person becomes sick, and remains more or less so, in spite of the remedies used, it would be wise to change to another room in the building, or to another house in the neighbourhood, or exposure; there are physical obstacles, and it is useless to contend against natural laws.

But a family may occupy a dwelling for a number of years in the enjoyment of general good health, when a change may occur, and one or more members, or all of them, may begin to complain, and may continue to be ailing, whatever may be done for restoration to health. Such changes are never without a sufficient cause. The rule should be in all cases where several members of a family are attacked with similar symptoms of sickness, to look about for a cause. Let the mind recur to any changes of any description. The last barrel of flour may have been largely adulterated with a heavy mineral substance, only to be detected by chloroform; a mill-pond may have been formed within a mile or two; or one may have been drained, and its former bottom exposed to a hot sun; a piece of swamp land may have been cleared; or a field may have been allowed to grow up with timber; or a belt of trees between the house and standing water or a sluggish stream may have been cut down, and thus the miasm which they absorbed is carried directly into the house; the well may have become foul; or a new well or spring way have been brought into use; any one of these, or of many other changes, is alone sufficient to make a whole family sickly. The first best step in all changes as to the health of a family for the worse, is to find out what changes have occurred of a physical character, and then seek to apply an appropriate remedy.

